# Anti-rheumatic property and physiological safety of KMU-11342 in in vitro and in vivo models

**DOI:** 10.1007/s00011-024-01904-6

**Published:** 2024-06-15

**Authors:** Hye Suk Baek, Victor Sukbong Hong, Hyunsu Kang, Sang-Jin Lee, Jin-Young Lee, Hyunju Kang, Seungik Jeong, Hyunho Jung, Jong Wook Park, Taeg Kyu Kwon, Chang-Nam Son, Sang Hyon Kim, Jinho Lee, Ki-Suk Kim, Shin Kim

**Affiliations:** 1 Department of Immunology, School of Medicine, Keimyung University, 1095 Dalgubeol-daero, Daegu, 42601 Republic of Korea; 2 Institute of Medical Science, School of Medicine, Keimyung University, 1095 Dalgubeol-daero, Daegu, 42601 Republic of Korea; 3 Institute for Cancer Research, School of Medicine, Keimyung University, 1095 Dalgubeol-daero, Daegu, 42601 Republic of Korea; 4 Department of Chemistry, Keimyung University, 1095 Dalgubeol-daero, Daegu, 42601 Republic of Korea; 5 R&D Center for Advanced Pharmaceuticals & Evaluation, Korea Institute of Toxicology, Daejeon, 34114 Republic of Korea; 6 Department of Physiology, Sungkyunkwan University School of Medicine, Suwon, 16419 Republic of Korea; 7 Department of Biological Sciences, Keimyung University, Daegu, 42601 Republic of Korea; 8 Department of Food and Nutrition, Keimyung University, Daegu, 42601 Republic of Korea; 9 New Drug Development Center, Daegu-Gyeongbuk Medical Innovation Foundation, Daegu, 41061 Republic of Korea; 10 Department of Rheumatology, Uijeongbu Eulji Medical Center, Eulji University School of Medicine, 712, Dongil-ro, Uijeongbu-si, 11759 Gyeonggi-do Republic of Korea; 11 Division of Rheumatology, Department of Internal Medicine, School of Medicine, Keimyung University, Daegu, 42601 Republic of Korea

**Keywords:** Rheumatoid arthritis, Protein kinase, Inflammation, NLRP3 inflammasome, Macrophage, Osteoclast differentiation

## Abstract

Rheumatoid arthritis (RA) is a chronic, systemic inflammatory disorder characterized by joint destruction due to synovial hypertrophy and the infiltration of inflammatory cells. Despite substantial progress in RA treatment, challenges persist, including suboptimal treatment responses and adverse effects associated with current therapies. This study investigates the anti-rheumatic capabilities of the newly identified multi-protein kinase inhibitor, KMU-11342, aiming to develop innovative agents targeting RA. In this study, we synthesized the novel multi-protein kinase inhibitor KMU-11342, based on indolin-2-one. We assessed its cardiac electrophysiological safety using the Langendorff system in rat hearts and evaluated its toxicity in zebrafish in vivo. Additionally, we examined the anti-rheumatic effects of KMU-11342 on human rheumatoid arthritis fibroblast-like synoviocytes (RA-FLS), THP-1 cells, and osteoclastogenesis in RAW264.7 cells. KMU-11342 demonstrated the ability to inhibit LPS-induced chemokine inhibition and the upregulation of pro-inflammatory cytokines, cyclooxygenase-2, inducible nitric oxide synthase, p-IKKα/β, p-NF-κB p65, and the nuclear translocation of NF-κB p65 in RA-FLS. It effectively suppressed the upregulation of NLR family pyrin domain containing 3 (NLRP3) and caspase-1 cleavage. Furthermore, KMU-11342 hindered the activation of osteoclast differentiation factors such as RANKL-induced TRAP, cathepsin K, NFATc-1, and c-Fos in RAW264.7 cells. KMU-11342 mitigates LPS-mediated inflammatory responses in THP-1 cells by inhibiting the activation of NLRP3 inflammasome. Notably, KMU-11342 exhibited minimal cytotoxicity in vivo and electrophysiological cardiotoxicity ex vivo. Consequently, KMU-11342 holds promise for development as a therapeutic agent in RA treatment.

## Introduction

Rheumatoid arthritis (RA) is characterized by joint damage stemming from the degradation of the extracellular matrix by infiltrating immune cells [1]. The pathogenesis of RA involves various cells, including activated macrophages, neutrophils, and synoviocytes. Moreover, chondrocytes and fibroblasts, T cells, and B cells, contribute to the production of chemokines and inflammatory cytokines, disrupting immune quilibrium and leading to damage in cartilage and bone [2]. Hence, RA’s emergence is attributed to complex and intertwined interactions among diverse cell types, though the precise mechanism is yet to be fully elucidated. Managing the overall inflammatory response of these immune cells is crucial in treating RA. Protein kinases pivotal in protein phosphorylation, and fundamental cellular regulatory processes, are significant targets in developing therapies for inflammatory and autoimmune diseases [3]. Drugs targeting various protein kinases have been formulated to treat autoimmune and inflammatory diseases, including RA. However, the disease’s intricate nature has led to variable and often unsatisfactory drug responses patients [4]. Therefore, developing effective new streatments for RA remains a formidable challenge.

In this study, we developed the novel multiple protein kinase inhibitor, KMU-11342, we assessed its electrophysiological safety and toxicity in vivo and ex vivo, and showcased its anti-rheumatic potential by revealing its anti-inflammtory mechanisms in vitro studies, in primary human RA fibroblast-like synoviocytes (RA-FLS) and by hindering osteoclastogenesis in RAW264.7 cells.

## Methods

### Experimental reagents

In this study, we utilized the following reagents and antibodies: Lipopolysaccharides (LPS) from *Escherichia coli* serotype 0111:B4 was acquired from Sigma-Aldrich (St. Louis, MO, USA, L4391). Recombinant soluble receptor activator of NF-κB (sRANKL) was obtained from PeproTech (Rocky Hill, NJ, USA, #315 − 11). Adenosine 5’-triphosphate disodium salt (ATP) and phorbol 12-myristate 13- acetate (PMA) were procured from Santa Cruz Biotechnology (# sc-202,040) and Sigma-Aldrich (St. Louis, MO, USA, P8139), respectively. Antibodies against TNF-α was purchased from abcam (Cambridge, UK, ab1793). Antibodies targeting phospho(p)-IKKα/β (#2697), p-Akt (8556 S), IKKα (#11,930), p-NF-κB p65 (#3033), NF-κB p65 (#4764), p-ERK (#9101), ERK (#9102), p-JNK (#9255), Akt (9272 S), p-TAK1 (9339 S), and NLRP3 (#15,101), were sourced from Cell Signaling Technology (Beverly, MD, USA). The anti-IL-1β (NB600-633) and iNOS (NB300-605) antibody were obtained from Novus Biologicals (Centennial, CO, USA). Antibodies for speck-like protein containing a CARD (ASC) (sc-22514R), NFATc-1 (sc-7294), c-Fos (sc-166,940), COX-1 (sc-70,877), NEK7 (sc-393,539), Caspase-1 (sc-56,036), IL-6 (sc-130,326), p-p38 (sc-7973), p38 (sc7972), JNK (sc-7345), MMP-9 (sc-6840), and TAK1 (sc-7967) were acquired from Santa Cruz. Biotechnology (Santa Cruz, CA, USA), COX-2 (SAB4200576) and The anti-β-actin antibody were purchased from Sigma-Aldrich (St. Louis, MO, USA). The anti-rabbit IgG- horseradish peroxidase (HRP), anti-horse and anti-mouse IgG-HRP antibodies were purchased from Santa Cruz Biotechnology (Santa Cruz, CA, USA). Pharmaceutical agent such as Celecoxib, Methotrexate, Ibrutinib and Tofacinib were obtained from Selleck Chemicals (Houston, TX, USA). Finally, XTT assay kit was procured from Welgene Inc. (Gyeongsan, Korea).

### Synthesis of KMU-011342

#### General information

All reactions were conducted using commercially available reagents and solvents, employed as received without further purification. Microwave-assisted reations were facilitated using a CEM Discover BenchMate. The Synthesized compounds underwent purification by flash column chromatography utilizing Merck Silica Gel 60 (230–400 mesh). Characterization of the compounds was achieved by ^1^H-NMR, employing a JEOL ECA 500 spectrometer (^1^H: 500 MHz, ^13^C: 125 MHz). Mass spectral analysis was conducted using a Waters ACQUITY UPLC, Micromass Quattro micro™ API.

#### 5-(4,4,5,5-Tetramethyl-1,3,2-dioxaborolan-2-yl)indolin-2-one (1)

In a microwave vessel, we combined 5-bromoindoline-2,3-dione (0.50 g, 2.2 mmol), hydrazine hydrate (0.14 g, 4.4 mmol), and ethanol (EtOH, 2.0 mL). The mixture underwent irradiation for 10 min at 100 °C with an applied power of 100 W. Following this, sodium hydroxide (0.18 g, 4.4 mmol), was added, and the mixture was again irradiated for 10 min at 80 °C with 100 W. Acidification was achieved using aqueous 2 M hydrochloric acid (HCl), and the resultant precipitate, formed upon the addition of cold water, was collected by filtration. The precipitate was washed with aqueous 2 M HCl and then with water, and dried in vacuo to yield 0.42 g of 5-bromoindolin-2-one. This intermediate was used in the suvsequent step without further purification. In a new microwave vessel, 5-bromoindolin-2-one (0.40 g, 1.9 mmol) and a mixture of 1,4-dioxane: EtOH (5:1) (2.4 mL), were combined. To this, bis(pinacolato)diboron (0.72 g, 3.8 mmol) and potassium acetate (KOAc; 0.56 g, 5.7 mmol), were added, and the reaction mixture was purged with N_2_ gas for 5 min. Subsequently, 1,1′-bis(diphenylphosphino)ferrocene]dichloropalladium(II) (PdCl_2_(dppf)) (0.069 g, 0.094 mmol) was introduced under a N_2_ atmosphere. The mixture was then irradiated for 10 min at 110 °C, applying 100 W. Post-irradiation, solvents were removed *in vacuo*, and the residue was treated with ethyl acetate (EA) and filtered through Celite. The filtrate was collected and solvents were evaporated *in vacuo*. The remaining residue was purified using silica gel chromatography with a hexane (Hex)/EA, mixture (1:1) to yield 0.41 g (82%) of the target compound. ^1^H-NMR (500 MHz, CDCl_3_) spectral data: δ 8.09 (s, 1H), 7.80–7.76 (m, 1H), 7.68 (s, 1H), 6.90 (d, *J* = 8.6 Hz, 1H), 3.54 (s, 2 H), 1.42 (s, 12 H).

#### 6-Chloro-*N*-cyclopentylpyrazin-2-amine (2)

Cyclopentylamine (0.40 mL, 4.1 mmol) and potassium carbonate (K_2_CO_3_; 0.93 g, 6.7 mmol) were combined in 5.0 mL of *N*,*N*-dimethylformamide, The mixture was stirred for 10 min at room temperature. Subsequently, 2,6-dichloropyrazine (0.50 g, 3.4 mmol), was added, and the reaction mixture was stirred overnight at room temperature. The solvent was then removed *in vacuo*. The residue underwent treatment with dichloromethane (DCM) and was filtered through Celite, The filtrate was collected and the solvent was removed *in vacuo*. The remaining residue was purified using silica gel chromatography with a 5:1 DCM/EA, which yielded 0.21 g (36%) of the title compound. ^1^H-NMR (500 MHz, CDCl_3_) spectral data: δ 7.76 (s, 1H), 7.72 (s, 1H), 4.74 (br, 1H), 4.09–4.03 (m, 1H), 2.10–2.03 (m, 2 H), 1.79–1.59 (m, 4 H), 1.49–1.43 (m, 2 H).

#### 5-(6-(Cyclopentylamino)pyrazin-2-yl)indolin-2-one (3)

In a microwave vessel compound **1** (0.39 g, 1.5 mmol), and compound **2** (0.20 g, 1.0 mmol), were combined with 1,4-dioxane (2.0 mL), EtOH (0.40 mL), and 2 M aqueous potassium carbonate (1.8 mL, 2.54 mmol). The mixture was purged with N_2_ gas for 10 min. Tetrakis(triphenylphosphine) palladium(0) (Pd(PPh_3_)_4_) (0.035 g, 0.030 mmol) was added under a N_2_ atmosphere. The reaction mixture was irradiated for 10 min at 110 °C, applying 100 W. Post-irradiation, the solvent was removed *in vacuo*. The residue was treated with DCM and filtered through Celite. The filtrate was collected and the solvent was removed *in vacuo*. The residue was then purified using silica gel chromatography with a DCM/EA 5:1 mixture, yielding 0.17 g (57%) of the target compound. ^1^H-NMR (500 MHz, DMSO-*d*_6_) spectral data: δ 10.54 (s, 1H), 8.16 (s, 1H), 7.93–7.86 (m, 2 H), 7.78 (s, 1H), 7.07 (d, *J* = 6.3 Hz, 1H), 6.90 (d, *J* = 9.2 Hz, 1H), 4.25–4.17 (m, 1H), 3.56 (s, 2 H), 2.07–1.92 (m, 2 H), 1.76–1.65 (m, 2 H), 1.65–1.53 (m, 2 H), 1.53–1.42 (m, 2 H).

#### (*Z*)-3-((1*H*-imidazol-5-yl)methylene)-5-(6-(cyclopentylamino)pyrazin-2-yl)indolin-2-one (KMU-011341)

A microwave vessel was loaded with compound **3** (0.080 g, 0.27 mmol), piperidine (5.0 µL, 0.051 mmol), 1*H*-imidazole-4-carbaldehyde (0.031 g, 0.32 mmol), and EtOH (2.0 mL). The reaction mixture was irradiated for 10 min at 80 °C, applying 100 W. The solvent was subsequently removed *in vacuo* and the residue was washed with water, DCM and methyl alcohol (MeOH). Drying under vacuum yielded 0.048 g (47%) of the title compound. ^1^H-NMR (500 MHz, DMSO-*d*_6_) spectral data: δ 11.19 (s, 1H), 8.37 (s, 1H), 8.30 (s, 1H), 8.03 (s, 1H), 8.00 (s, 1H), 7.97 (d, *J* = 8.0 Hz, 1H), 7.83 (s, 1H), 7.70 (br, 1H), 7.09 (d, *J* = 6.3 Hz, 1H), 7.01 (d, *J* = 8.0 Hz, 1H), 4.34–4.19 (m, 1H), 2.18–1.91 (m, 2 H), 1.82–1.67 (m, 2 H), 1.67–1.57 (m, 2 H), 1.57–1.32 (m, 2 H); ^13^C-NMR (125 MHz, DMSO-*d*_6_) spectral data: δ 170.74, 154.43, 148.97, 141.15, 140.33, 139.19, 131.43, 131.22, 128.70, 127.41, 127.13, 125.38, 124.02, 120.37, 117.77, 110.54, 52.25, 32.97, 24.15; MS(ESI) calcd. Analysis: calculated for [C_21_H_20_N_6_O + H]^+^ = 373.2 found 373.

### Isolating and culturing fibroblast-like synoviocytes from primary human rheumatoid arthritis (RA-FLS)

RA-FLS were sourced from the Division of Rheumatology, Department of Internal Medicine, under the supervision of Dr. Chang-Nam Son at Keimyung University. Primary cell isolation was conducted using tissues obtained from patients undergoing bone replacement surgery to address damage incurred during the procedure, specifically those with osteoarthritis. All patients provided prior informed consent for the utilization of their tissues, and the study was conducted in accordance with the guidelines of Keimyung University Dongsan Hospital [IRB No. 2020-11-031], as approved by the Keimyung Academic Review Board. Tissues, encompassing fat, ligaments, and cartilage, were meticulously dissected by skilled technicians within the institution. Subsequently, the dissected tissues were placed in a 100-mm dish filled with cell-culture phosphate-buffered saline (PBS) and thoroughly cleansed with DMEM (Welgene, Gyeongsan, Korea). The osteoarthritic tissues were then carefully crushed into 2–3 mm pieces, and 4 mg/mL type-1 collagenase (Worthington Biochemical, Freehold, NJ, USA) was introduced, followed by incubation under 5% CO_2_ at 37 °C for 4 h. After centrifugation (1,200 rpm, 10 min), the isolated cells were resuspended in DMEM supplemented with 10% FBS, 2 mmol/L glutamine, penicillin (100 U/mL), and streptomycin (100 mg/mL), and subsequently placed in rotation within a 75 cm^2^ flask. Following a day, the adherent cells were maintained in a controlled environment of 5% CO_2_ at 37 °C in DMEM containing 10% FBS. The culture medium was replenished every three days. Upon reaching 90 – 95% confluency, the medium was diluted at a 1:3 ratio with fresh culture medium, and the cells were serially cultivated before being preserved in a nitrogen tank.

### The isolated heart preparation of Langendorff

Sprague Dawley (SD) male rats (200–250 g) received an intravenous injection of 1000 IU kg − 1 heparin followed by anesthesia administered via an intravenous injection of 30 mg kg − 1 sodium pentobarbital. Once the pedal reflex was absent, their hearts were swiftly excised and perfused through the aorta on a Langendorff apparatus (EMKA Technologies, Paris, France) utilizing modified Krebs–Henseleit buffer saturated with carbogen (95% O_2_ and 5% CO_2_) containing 112 mM NaCl, 5 mM KCl, 11.5 mM glucose, 25 mM NaHCO_3_, 1.2 mM MgSO_4_, 1.2 mM KH_2_PO_4_, 2 mM pyruvic acid, and 1.25 mM CaCl_2_ The perfusion was maintained at a constant pressure (60–80 mmHg).

### Left ventricle pressure (LVP) and electrocardiogram (ECG) recordings

LVP, was measured by placing a water-filled latex balloon attached to a metal cannula, inside the left ventricle via the pulmonary vein, which was then connected to a pressure transducer (EMKA Technologies). ECG readings were taken using two surface electrodes (EMKA Technologies) positioned against the epicardium. One electrode was placed on the right ventricle near the atrium-ventricle ring, and the other on the left ventricle. All hemodynamic parameters were captured and analyzed using iox2 software (EMKA Technologies) for a duration of 10–15 min before and after reperfusion with varying concentrations of KMU-11342 in the buffer. QTc intervals in milliseconds were computed using Bazett’s formula (QTcB = QT/RR^1/2^, Fridericia: QTcF = QT/RR^1/3^). KMU-11342 administered in escalating concentrations from 0.1 to 5 µM. The study protocols were reviewed and sanctioned by the Institutional Animal Care and Use Committee (IACUC) of the Korea Institute of Toxicology (KIT) under the code RS21006.

### Toxicology test in zebrafish

Zebrafish (Danio rerio) were sourced from the Zebrafish International Resource Center (University of Oregon, Eugene, OR, USA) and maintained in accordance with the guideline [5]. In briefly, the fish were fed standard diets of feed, and raw brine substituted shrimp. These fish were cultured in UV-treated water under the following conditions. The aquatic environment was strictly regulated: temperature, at 28.5 °C ± 1 °C; pH, 6.9–7.5; and a circadian, rhythm of a 14 h light/10 h dark cycle. Post natural mating, zebrafish embryos were collected rinsed thoroughtly, and carefully inspected. The embryos were then exposed to designated concentrations of KMU-11342 at 24 h- post fertilization, HPF. KMU-11342’s toxicity was assessed based on morphological changes in the embryos, employing a modified version of the fish embryo acute toxicity (FET) test (OECD Guideline 236 (No, 2013)). Paramenters such as hatchability, body length, average heart rate per minute, and the visual appearance of treatment and solvents were meticulously analyzed. Photographic documentation was performed using a light microscope (Carl Zeiss Stereo microscope DV4, Seoul, Republic of Korea) to accurately quantify viable zebrafish.

### Cell cultivation and osteoclastogenesis

Cell cultures were maintained continuously throughout the duration of experiment. The THP-1 cell line, consisting of human monocytic leukemia cells, and the RAW264.7 cell line, comprising murine macrophage cells, were the primary cells used in this study. THP-1 cells were plated at densities tailored to each specific experiment and incubated for 24 h with 100 nM PMA in the medium. Both cell lines, THP-1 and RAW264.7 were obtained from the Korea Cell Line Bank (Seoul, Korea) and cultured in Roswell Park Memorial Institute (RPMI1640; Welgene, Gyeongsan, Korea) and Dulbecco’s Modified Eagle Medium (DMEM; GIBCO, BRL, Grand Island, NY, USA) respectively. The culture medium was supplemented with a 1% antibacterial-antifungal solution (GIBCO, BRL, Grand Island, NY, USA) and 10% fetal bovine serum (FBS; GIBCO, BRL, Grand Island, NY, USA) containing of penicillin and streptomycin. Conditions within the incubator were regulated at 95% humidity and 37 °C, with a constant 5% CO_2_. Cells were sub-cultured weekly or bi-weekly. For the exploration of osteoclast differentiation, RAW264.7 cells were seeded at density of 4 × 10^4^ cells/well in 12-well plates. Differentiation was induced over a 5-day period with α-minimum essential medium (MEM; Welgene, Gyeongsan, Korea) supplemented with 50 ng/mL RANKL. The culture medium was refreshed every three days. Groups undergoing treatment were pre-treated with KMU-11342 at concentrations of 0.01, 0.25, and 0.5 µM for 1 h prior to RANKL administration.

### Kinase-profiling analysis

The kinase inhibitor KMU-11342, underwent kinase-profiling at Eurofins Cerep S.A. (Celle-Lévescault, France). The profiling was conducted in alignment with the manufacturer’s established protocol. In the analyses of individual kinase and their substrates, the compound was used at a concentration of 1 µM/mL, alongside ATP at the Km value. In brief, each kinase assay involbed a reaction mixture comprising 8 mM/L MOPS at pH 7.0, 0.2 M EDTA, 50 µM/L EAIYAAPFAKK, and 10 mM/L acetate. [Υ-33P] ATP was incorporated tailored to specific activity and concentration requirements, was introduced, and the ensuing response to the added Mg/ATP mixture was precisely measured. The reaction was allowed to proceed at room temperature for 40 min. Subsequently, phosphoric acid 0.5% was introduced to halt the reaction. Then, 10 µL of the reaction mixture was applied to filter paper, which was sequentially rinsed with methanol and phosphoric acid, followed by drying. The dried samples were subjected to scintillation counting for 4 min, utilizing a P30 filter matrix.

### Measurement of cytotoxicity

The intracellular toxicity induced by the synthetic compound KMU-11342 was scrutinized through the 2,3-bis-(2-methoxy-4-nitro-5-sulfophenyl)-2 H-tetrazolium-5-carboxanilide (XTT) assay. THP-1 and RAW264.7 cells (1 × 10^5^ cells) were seeded in 96-well plates, treated with varying concentrations of KMU-11342, and incubated in a controlled culture environment at 37 °C for 24 h. Following this incubation period, the culture solution was augmented with XTT solution, and cell survival was evaluated on the subsequent day. The XTT, utilized at a concentration of 0.5 mg/mL, underwent reaction under identical culture conditions for 2 h. Post-reaction, the absorbance of the supernatant was quantified at 450 nm.

### Analysis of western blotting

To validate the modulation of expression levels in inflammation-related signaling proteins elicited by KMU-11342, Western blotting was systematically conducted. RA-FLS and THP-1 cells were seeded at densities of 1 × 10^6^ cells/well, and 2 × 10^6^ cells/well, respectively, in 100 mm and 60 mm dishes. Following a 24 h culture period, the cells were treated with KMU-11342 at concentrations of 0.1, 0.5, and 1 µM/mL for 1 h, and then stimulated with LPS for 6 h. Total protein was extracted from the treated using RIPA buffer solution (Cell Signaling, Technology, Beverly, MD, USA). Protein concentrations were determined using a bicinchoninic acid (BCA) assay kit (Thermo Scientific, Wilmington, DE, USA). The supernatants of the cell culture media were precipitated using a trichloroacetic acid (TCA), according to the protocol by Luis Sanchez (2001). After the precipitation step, SDS-PAGE was performed, wherein the samples were dissolved in 20 µL of 2× or 4× sample buffer (with or without β-mercaptoethanol), followed by boiling for 10 min at 95 °C before loading onto a polyacrylamide gel. Equal amounts of protein were subjected to electrophoresis in 10% SDS-polyacrylamide gels and subsequently transferred to nitrocellulose membranes. Following a blocking step with 5% skim milk for 1 h, the membranes were incubated for 24 h with appropriately diluted primary antibodies specific to the target proteins of interest. Post-incubation, the membranes were washed with Tris-buffered saline/Tween buffer and then subjected to a 1 h incubation at room temperature with HRP-conjugated anti-IgG secondary antibodies. Protein bands were visualized using enhanced chemiluminescence, and the signal intensity was quantified with a Chemi Image System (Fusion Fx7, Vilber Lourmat, Collégien, France). Notably, β-actin served as the internal control for the Western blot analysis.

### RNA isolation and quantitative real time PCR

To analyze gene expression related to pro-inflammatory cytokines and chemokines at the mRNA level through real-time quantitative PCR (qPCR). THP-1 cells were cultured by uniformly seeding a consistent cell count in 6-well dishes, each receiving an identical number of cells, and treated with KMU-11342 at concentrations of 0.1, 0.5, and 1 µM/mL for 1 h. Subsequently, the cells were stimulated with LPS for a duration of 6 h. Total RNA was extracted from each sample using TRIzol solution (Invitrogen, San Diego, CA, USA), following the manufacture’s protocol. Subsequently, 1 µg of the extracted and quantified RNA underwent reverse transcription, employing deoxynucleoside triphosphate (dNTP), buffer, dithiothreitol, RNase inhibitor, and SuperScript II reverse transcriptase. The resultant cDNA was subjected to RT-PCR using specific primers, and the outcomes were analyzed. The primers utilized in this study are detailed in Table 1.

**Table 1:** Primer sequences for Real time PCR

Primers	Sequences (5’ → 3’)	
Human primer		
CXCL10	Forward	GTG CGA TTC AAG GAG TAC CTC
	Reverse	TGA TGG CCT TCG ATT CTG GAT T
CCL2	Forward	CAG CCA GAT GCA ATC AAT GCC
	Reverse	TGG AAT CCT GAA CCC ACT TCT
CCL3	Forward	AGT TCT CTG CAT CAC TTG CTG
	Reverse	CGG CTT CGC TTG GTT AGG AA
CCL4	Forward	CTG TGC TGA TCC CAG TGA ATC
	Reverse	TCA GTT CAG TTC CAG GTC ATA CA
iNOS	Forward	CTG TCT GGT TCC TAC GTC ACC
	Reverse	CCC ACG TTA CAT GGG AGG ATA
COX-2	Forward	ATC ACA GGC TTC CAT TGA CC
	Reverse	TAT CAT CTA GTC CGG AGG GG
IL-1β	Forward	CCT TGG GCC TCA AGG AAA A
	Reverse	CTC CAG CTG TAG AGT GGG CTT A
TNF-α	Forward	GGA GAA GGG TGA CCG ACT CA
	Reverse	CTG CCC AGA CTC GGC AA
IL-6	Forward	ATG GCA CAG TAT CTG GAG GAG
	Reverse	TAA GCT GGA CTC ACT CTC GGA
β-actin	Forward	AAT CTG GCA CCA CAC CTT CTA
	Reverse	ATA GCA CAG CCt GGA TAG CAA
Mouse primer		
Cathepsin K	Forward	CAG CAG AAC GGA GGC ATT GA
	Reverse	CCT TTG CCG TGG CGT TAT AC
TRAP	Forward	AAG GCG AGA GAT TCT TTC CCT G
	Reverse	ACT GGG GAC AAT TCA CTA GAG C
NFATc1	Forward	GGG TCA GTG TGA CCG AAC AT
	Reverse	GGA AGT CAG AAG TGG GTG GA
MMP-9	Forward	GCC CTG GAA CTC ACA GCA CA
	Reverse	TTG GAA ACT CAC ACG CCA GAA G
OSCAR	Forward	AGG GAA ACC TCA TCC GTT TG
	Reverse	GAG CCG GAA ATA AGG CAC AG
β-actin	Forward	ATT TCT GAA TGG CCC AGG T
	Reverse	CTG CCT CAA CAC CTC AAC C
PGC-1α	Forward	AAGCTGAAGCCCTCTTGCAA
	Reverse	ACTGTACCGGGCCCTCTTG
PGC-1β	Forward	CGCTCCAGGAGACTGAATCCAG
	Reverse	CTTGACTACTGTCTGTGAGGC
CS	Forward	AATCAGGAGGTGCTTGTC
	Reverse	GTAGTCTCGTAACTTCTCATCT
TFAM	Forward	CACCCAGATGCAAAACTTTCAG
	Reverse	CTGCTCTTTATACTTGCTCACAG
16 S rRNA	Forward	CCGCAAGGGAAAGATGAAAGAC
	Reverse	TCGTTTGGTTTCGGGGTTTC
Hexokinase 2	Forward	GCCAGCCTCTCCTGATTTTAGTGT
	Reverse	GGGAACACAAAAGACCTCTTCTGG
Cytochrome B	Forward	CATTTATTATCGCGGCCCTA
	Reverse	TGTTGGGTTGTTTGATCCTG

### Immunofluorescence staining

RA-FLS and THP-1 cells (1 × 10^3^) were cultured on 8-chamber glass slides for 24 h. The cells were then treated with KMU-11342 at concentrations of 0.1, 0.5, and 1 µM/mL for 1 h, followed by LPS stimulation for 6 h. The cells were meticulously washed with PBS, fixed using 4% formaldehyde, and permeabilized with 0.2% Triton X-100 in PBS. To block nonspecific binding, the cells were incubated with bovine serum albumin (BSA) for 1 h. Thereafter, they were incubated with primary antibodies at 4°C for 24 h. Subsequent steps involved incubation with fluorescein isothiocyanate-conjugated goat anti-mouse IgG (Thermo Scientific, Wilmington, DE, USA) and 4’-6-diamidino-2-phenylindole (DAPI) for nuclear staining (Vector Laboratories, Burlingame, CA, USA). Finally, the stained cells were meticulously examined under a fluorescence microscope to capture and analyze the immunofluorescence patterns.

### Tartrate-resistant acid phosphatase staining (TRAP) and F-actin ring staining

RAW264.7 cells were seeded in 12-well culture dishes and treated with 50 ng/mL RANKL or pre-treated with KMU-11342 (at concentrations of 0.01, 0.1, and 0.25 µM/mL) for 1 h. Following a five-day cultivation period, the cells underwent TRAP staining using a leukocyte acid phosphatase kit (TaKaRa. Bio, Kyoto, Japan) follosing the manufacturer’s instructions. The cultured cells were fixed in a fixation solution for 20 min at room temperature and subsequent washing with distilled water. Microscopic examination identified cells with three or more nuclei as multinuclear osteoclasts. For F-actin ring staining, RAW264.7 cells were again plated in a 12-well culture dish and stimulated with 50 ng/mL RANKL for 6 days. Staining was accomplished using Phalloidin CruzFluor™488 Conjugate (Santa Cruz, CA, USA). Following a wash with PBS, cells were fixed in 4% neutral buffered formalin for 20 min at room temperature. Subsequent steps involved cell permeabilization for 10 min with 0.1% Triton X-100 in PBS, blocking with 1% BSA for 1 h at room temperature, and incubation with the stain antibody (1:5000 dilution) for 30 min. After triple washing with PBS, the stained cells were captured through imaging using a fluorescence microscope.

### Bone resorption assay

The osteoclasts were generated using the forementioned method and cultured on Osteo Assay Surface multi-well plates (CSR-BRA-24KT, Cosmo Bio co. LTD, Japan) for 7 days. Various concentrations of KMU-11342 were introduced to the cultures from day 1. After 7 days, the plates were washed with saline and a solution of 5% sodium hypochlorite solution was applied for 5 min to detach the cells. The resorption areas were visualized under a microscope. Comparative analysis of each group was performed using ImageJ software.

### Mitochondrial bioenergetics assay

Mitochondrial respiration and extracellular acidification rate (ECAR) were assessed using the Seahorse XF96e Extracellular Flux analyzer (Seahorse Biosciences, North Billerica, MA, USA). RAW264.7 cells were pretreated with KMU-11342 for 1 h, stimulated with 100 ng/mL LPS for 2 h, and then treated sequentially in the following sequence: The Oxygen consumption rate (OCR) was then measured after sequentially after the addition of 1 µM oligomycin (complex V inhibitor) at baseline, 0.5 µM, and 1 µM carbonyl cyanide 4-(trifluoromethoxy) phenylhydrazone (fCCP, electron transport chain separator), and ultimately 1 µM rotenone (complex III inhibitor) and 1 µM antimycin A (complex I inhibitor). OCR measurements were taken in 8 wells for each condition at three distinct time points across the four evaluation. Alternatively, an XF cell mito stress test (Agilent Technologies) was performed according to the manufacturer’s instructions. All OCR data were normalized to total protein per well measured using the Bradford assay.

### Mitochondrial DNA copy number

Total DNA was extracted from RAW264.7 cell samples using the DNeasy Blood and Tissue kit (Qiagen, Valencia, CA, USA), according to the manufacturer’s instructions. Analysis of mtDNA content to obtain the mtDNA/nDNA ratio was used the real time qPCR. Different genes are used to evaluate the relative copy number of mtDNA and nDNA. We determined mitochondrial genes using 16 S ribosomal RNA and cytochrome b, and the nuclear encoded genes using hexokinase 2.

### Statistical analysis

Disparities among the groups were meticulously scrutinized employing Student’s two-tailed t-test. The examination of experimental outcomes was executed utilizing the GraphPad Prism 5.0 program (GraphPad Software, San Diego, CA, USA). To discern variations between each group, a one-way analysis of variance was applied, and the significance between control and experimental groups was assessed at a threshold of *p* < 0.05, employing Tukey’s multiple comparison test (* *p* < 0.05, ** *p* < 0.01, # *p* < 0.001).

## Results

### Cardiotoxicity and safety profiling of a multi-protein kinase inhibitor, KMU-11342 in zebrafish in vivo and rat heart ex vivo model

**Fig. 1:**
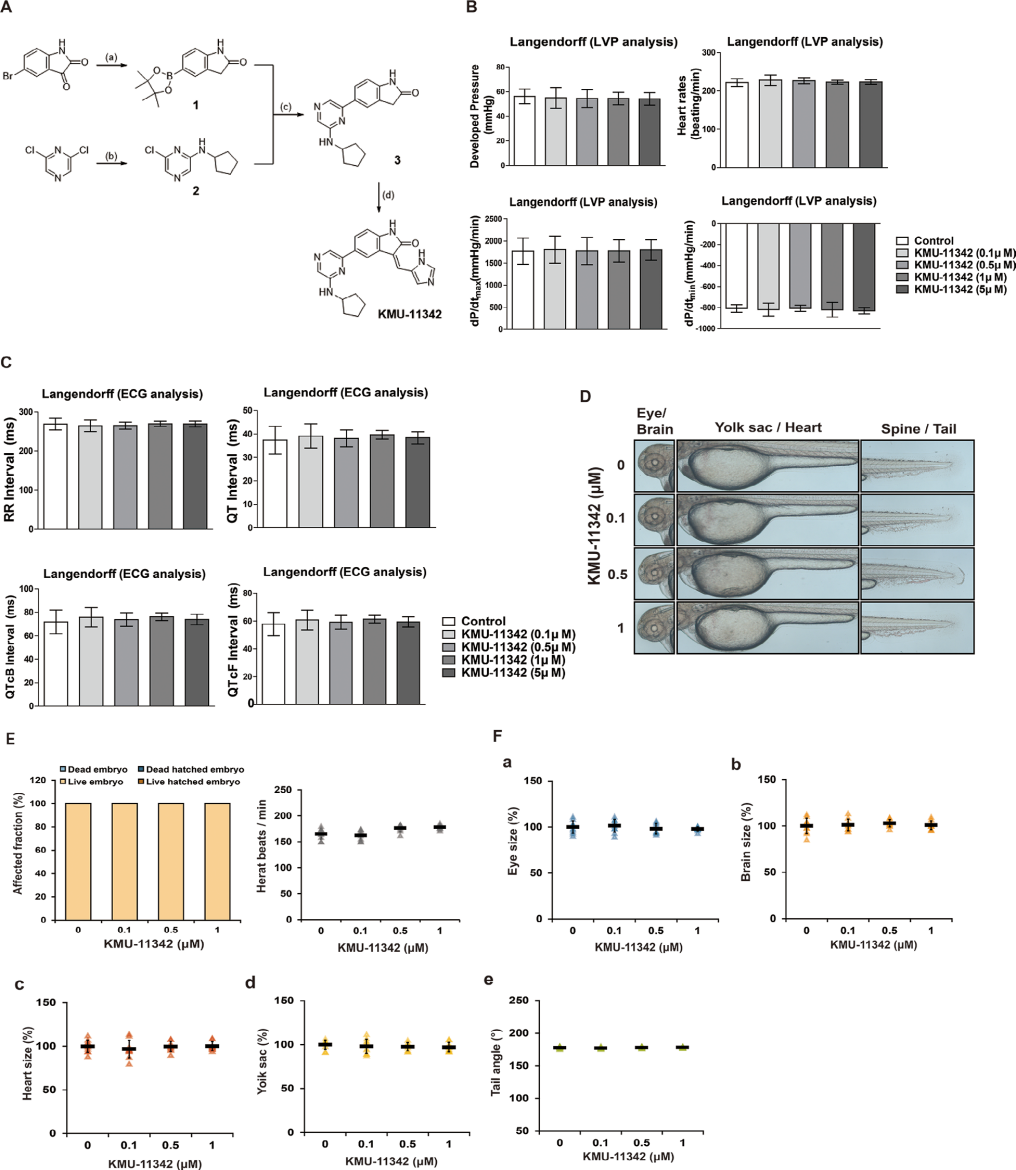
Discovery and the safety profiling of KMU-11342. (**A**) Chemical structure and synthesis of KMU-11342. Reagents and experimental conditions: (**a**) i) NH_2_NH_2_, NaOH, EtOH, microwave; ii) bis(pinacolato)diboron, PdCl_2_(dppf), KOAc, 1,4-dioxane: EtOH (5:1), microwave; (**b**) K_2_CO_3_, cyclopentylamine, DMF; (**c**) PdCl_2_(PPh_3_)_2_, 2 M Na_2_CO_3_, 1,4-dioxane: EtOH (5:1), microwave; (d) piperidine, 1 H-imidazole-4-carbaldehyde, EtOH, microwave. (**B, C**) All experiments were conducted after stabilizing for 10 min and starting. Next, the cells were treated with KMU-11342 in a dose-dependent manner (0.1, 0.5, 1, 5 µM) by perfusion. (**B**) Effect of KMU-11342 on left ventricle pressure analysis. (**C**) Effect of KMU-11342 on electrocardiogram analysis. Values in the graph (**B, C**) indicate the mean ± SD of three independent experiments. (**D**) Effect of KMU-11342 on the morphological characteristics of the developing zebrafish embryo. Zebrafish embryos were treated with KMU-11342 at the indicated concentrations for 24 h. Magnified image of the anterior-median-posterior region of a zebrafish larvae. (**E**) Affected fraction and heart beats per minute, (**F-a**) relative size of the eye, (**b**) relative brain volume in the anterior area, (**c**) Heart size, (**d**) edema in the yolk sac, (**e**) Tail angel

KMU-11342 was synthesized from 5-bromoisatin as shown in Fig. 1A. Compound **1** was derived through reduction and Miyaura borylation reaction, and its Suzuki coupling with the pyrazine derivative, **2**, yielded compound **3**. The final product, KMU-11342, was formed via Knoevenagel condensation of compound **3** with 1*H*-imidazole-4-carbaldehyde.

We further investigated the inhibitory effect of KMU-11342 on inflammatory kinases. Kinase panel screening demonstrated that KMU-11342 inhibited the activities of various kinases, including IKKα(h), IKKβ(h), JAK1(h), JAK2(h), JAK3(h), MAPK1(h), JNK3(h), TAK1(h), and TYK2(h) with percentages of inhibition at 1 µM concentration being 98, 53, 76, 79, 99, 97, 94,71, and 93%, respectively (Table 2). These results underscore KMU-11342’s potent inhibitory activity against inflammatory protein kinases. As drug-induced cardiotoxicity is a significant factor in the discontinuation or withdrawal of commercial drugs from the market [6], in vivo LVP assays are crucial for ensuring cardiac safety during drug development [7]. To evaluate the potential cardiotoxicity of KMU-11342, we employed the langendorff heart system for LVP, and electrocardiogram (ECG) analysis. Our analysis of cardiotoxicity factors including heart rate, developed pressure, maximal rates of contraction and relaxation (dP/dtmax and dP/dtmin), and ECG waveform parameters RR-interval (RR-I), QT-interval (QT-I), QT correction Bazett (QTcB-I) and Fridericia (QTcF-I), revealed that KMU-11342 did not affect any hemodynamic parameters in LVP analysis (Fig. 1B). Moreover, ECG analysis did not indicate any dose dependent cardiotoxicity induced by KMU-11342 (Fig. 1C). We also assessed the toxicity of KMU-11342 in zebrafish embryos. Observations showed no significant alterations in key phenotypes of embryos exposed to KMU-11342 up to a concentration of 1 µM. Image analysis demonstrated no changes in brain growth, eye size, yolk sac swelling, or dorsal curvature of the body axis in a dose-dependent manner with KMU-11342 (Fig. 1D). Additionally, post-treatment with KMU-11342, there were no noticeable effects such as delayed hatching, heartbeat pattern alterations, or developmental anomalies (Fig. 1E). Treatment with KMU-11342 at concentrations of 0.1, 0.5, and 1 µM had no impact on heartbeats, eye size, brain size, heart size, embryonic yolk swelling, or tail angle (Fig. 1Fa-e). Collectively, these findings suggest that KMU-11342 exhibits minimal toxicity, inclusive of cardiotoxicity and developmental toxicity.

**Table 2:** Kinase activity of 37 protein kinases upon treatment with 1µM KMU-11342

KMU-11342 1µM	
Kinase	Activity(% Control)*
Blk(h)	9
Bmx(h)	12
BTK(h)	17
cSRC(h)	33
Flt3(h)	2
Fgr(h)	31
Fyn(h)	19
Hck(h)	8
IKKα(h)	2
IKKβ(h)	47
Itk(h)	19
JAK1(h)	24
JAK2(h)	21
JAK3(h)	1
JNK1α1(h)	70
JNK2α2(h)	88
JNK3(h)	16
Lck(h)	10
Lyn(h)	13
MAPK1(h)	3
MAPKAP-K2(h)	96
MKK4(m)	55
MKK6(h)	57
MSK1(h)	56
PKA(h)	70
ROCK-II(h)	4
Pyk2(h)	82
SAPK2a(h)	55
SAPK3(h)	85
SAPK4(h)	45
Syk(h)	89
TAK1(h)	29
Tec(h) activated	17
Txk(h)	2
TYK2(h)	7
Yes(h)	10
ZAP-70(h)	86

### KMU-11342 attenuated LPS-induced activation of MAPKs, cyclooxygenase-2 (COX-2), and inducible nitric oxide synthase (iNOS) pathway in human RA-FLS and THP-1 cells

**Fig. 2:**
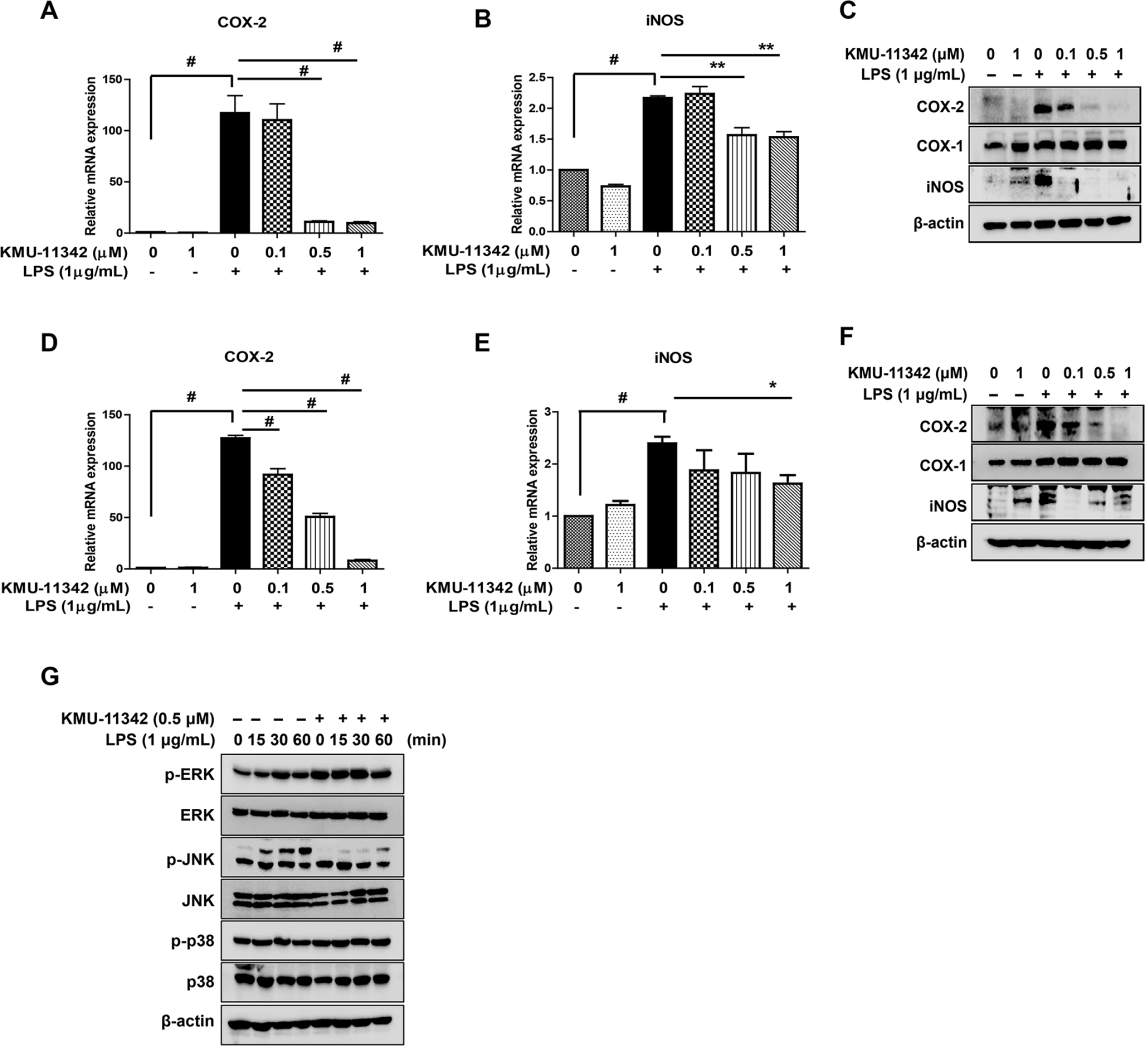
Inhibitory effect of KMU-11342 on LPS-induced up-regulation of COX-2 and iNOS expression in human RA-FLS and THP-1 cells. (**A, B**) In human RA-FLS; total RNA was extracted, and used to evaluate the mRNA expression levels of COX-2 and iNOS, respectively ** *p* < 0.01, # *p* < 0.001 compared with LPS alone treated group. (**C**) Whole cell lysates were isolated and used to measure the protein expression levels of COX-1, COX-2 and iNOS by Western blotting. (**D, E**) In THP-1 cells; total RNA was extracted, and used to evaluate the mRNA expression levels of COX-2, respectively. * *p* < 0.05 # *p* < 0.001 compared with LPS alone treated group. (**F**) Whole cell lysates were isolated and used to measure the protein expression levels of COX-1, COX-2 and iNOS by Western blotting. (**G**) THP-1 cells were stimulated with LPS (1 µg/mL) for the indicated time points. Whole cell lysates were isolated and used to measure the protein expression levels of p-ERK, ERK, p-JNK, JNK, p-p38, and p38 by Western blot analysis. Values in the graph (**A, B, D, E**) indicate the mean ± SD of three independent experiments

COX-2 and iNOS are pivotal in mediating inflammatory response. The pre-treatment with KMU-11342 curtailed the LPS-induced up-regulation of COX-2 and iNOS in both human RA-FLS and THP-1 cells (Fig. 2). However, KMU-11342 did not suppress the protein expression level of COX-1 (Fig. 2C, F), indicating a selective inhibitory effect of KMU-11342 on COX-2. Subsequently, we explored whether KMU-11342 could inhibit MAPKs, which are upstream regulators of the pro-inflammatory cytokines, COX-2, and iNOS. The results indicated that KMU-11342 effectively inhibited the LPS-stimulated activation of JNK, but not of ERK and p38, in THP-1 cells (Fig. 2G). These findings underscore the potential of KMU-11342 in selectively modulating key inflammatory pathways, offering insights into its therapeutic relevance in mitigating inflammation-associated responses.

### KMU-11342 inhibits LPS-stimulated inflammatory mediator production in human RA-FLS and THP-1 cells

**Fig. 3:**
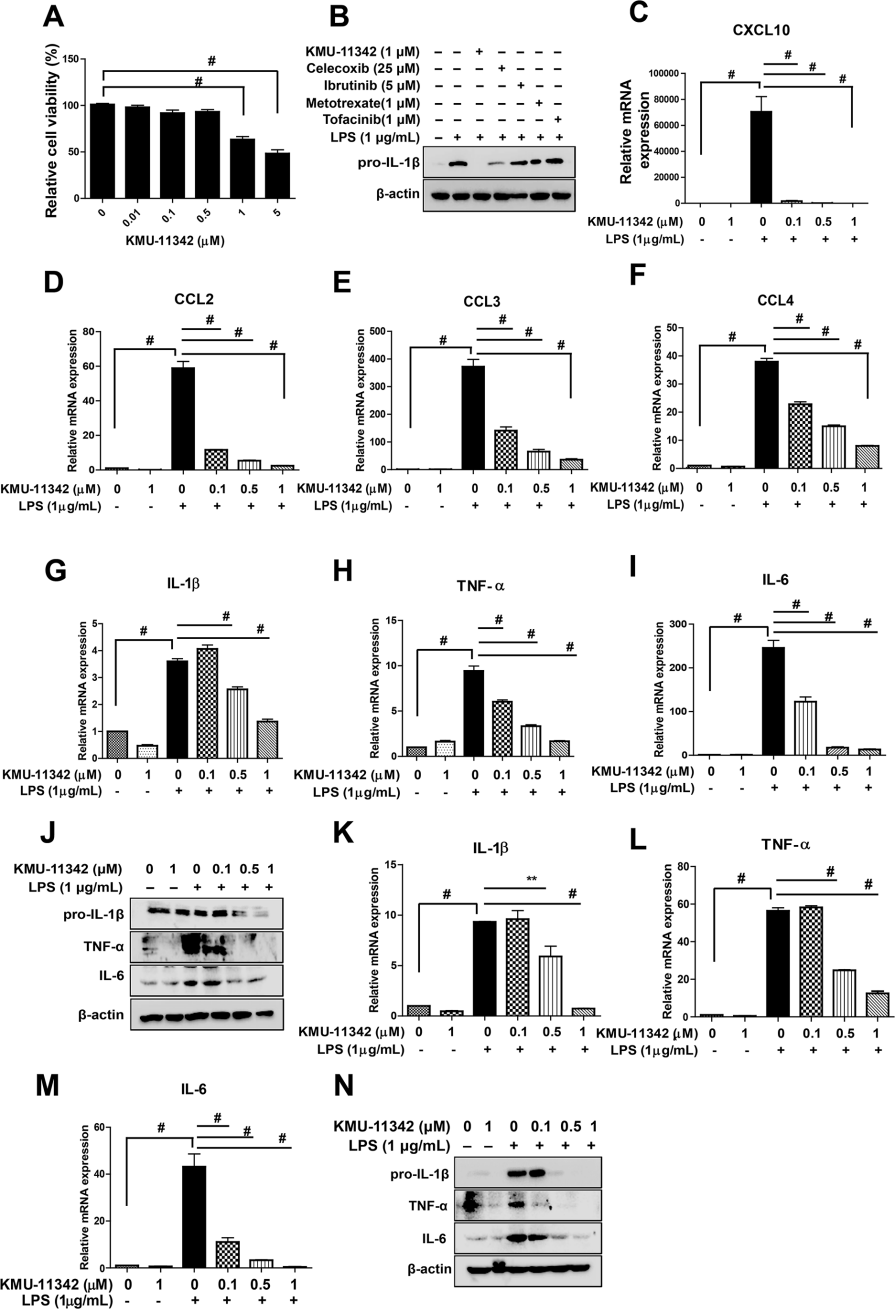
Inhibitory effect of KMU-11342 on LPS-mediated up-regulation of inflammatory mediators in human RA-FLS and THP-1 cells. (**A**) Cytotoxic effect of KMU-11342 in THP-1 cells was performed by XTT assay. # *p* < 0.001 compared with control group. Cell were differentiated into macrophages for 24 h using PMA (100 nM), then the cells were treated with different doses of KMU-11342 (0.01, 0.1, 0.5, 1, 5, and 10 µM) for 24 h. (**B**) THP-1 cells were differentiated into macrophages for 24 h using PMA (100 nM), and treated with LPS (1 µg/mL) for 6 h after pre-treatment with KMU-11342 (1 µM), celecoxib (25 µM), ibrutinib (5 µM), methotrexate (1 µM) and tofacinib (1 µM) for 1 h. Whole cell lysates were isolated and used to measure the protein expression levels of pro-IL-1β by Western blot analysis. (**C–F**) Human RA-FLS cells were treated with LPS (1 µg/mL) for 6 h after pre-treatment with different doses of KMU-11342 (0.1, 0.5, and 1 µM) for 1 h. Total RNA was extracted, and used to evaluate the mRNA expression levels of CXCL10, CCL2, CCL3, and CCL4, respectively. # *p* < 0.001 compared with the LPS only group. (**G–N**) Human RA-FLS and THP-1 cells were pre-treatment with the indicated concentrations of KMU-11342 (0.1, 0.5, and 1 µM) for 1 h and stimulated with LPS (1 µg/mL) for 6 h. (**G–I**) In human RA-FLS; total RNA was extracted, and used to evaluate the mRNA expression levels of IL-1β, TNF-α and IL-6, respectively # *p* < 0.001 compared with LPS alone treated group. (**I**) Whole cell lysates were isolated and used to measure the protein expression levels of IL-1β, TNF-α and IL-6 by Western blotting. (**J–M**) In THP-1 cells; Total RNA was extracted, and used to evaluate the mRNA expression levels of IL-1β, TNF-α and IL-6, respectively ** *p* < 0.01, # *p* < 0.001 compared with LPS alone treated group. (**N**) Whole cell lysates were isolated and used to measure the protein expression levels of IL-1β, TNF-α and IL-6 by Western blotting. Values in the graph (**A, C–I, K–M**) indicate the mean ± SD of three independent experiments

To assess the potential cytotoxic effects of KMU-11342 in THP-1 cells, an XTT assay was conducted. The results indicated that KMU-11342 exhibited no cytotoxicity at concentrations up to 1 µM (Fig. 3A). Further, to gauge the potential of KMU-11342 as an RA therapeutic, we compared its anti-inflammatory activity with various other RA and anti-inflammatory agents noting the potential superiority of KMU-11342. Specifically, THP-1 cells pre-treated with 1 µM KMU-11342 exhibited inhibited LPS-induced up-regulation of IL-1β, a response not observed even at higher concentrations of other drugs tested (Fig. 3B). Additionally, KMU-11342 exhibited a significant inhibitory effect on the LPS-stimulated up-regulation of chemokine mRNA levels, including CXCL10, CCL2/MCP-1, CCL3/MCP-1α, and CCL4/MCP-1β, in Human RA-FLS (Fig. 3C-F). Further investigation confirmed the anti-inflammatory efficacy of KMU-11342 was undertaken. Pre-treatment of both human RA-FLS and THP-1 cells with KMU-11342 resulted in the inhibition of LPS-induced up-regulation of pro-inflammatory cytokines such as IL-1β, TNF-α, and IL-6 (Fig. 3G–N).

### KMU-11342 attenuated of LPS-induced activation of the TAK1-NF-κB-NLRP3 inflammasome pathway in in human RA-FLS and THP-1 cells

**Fig. 4:**
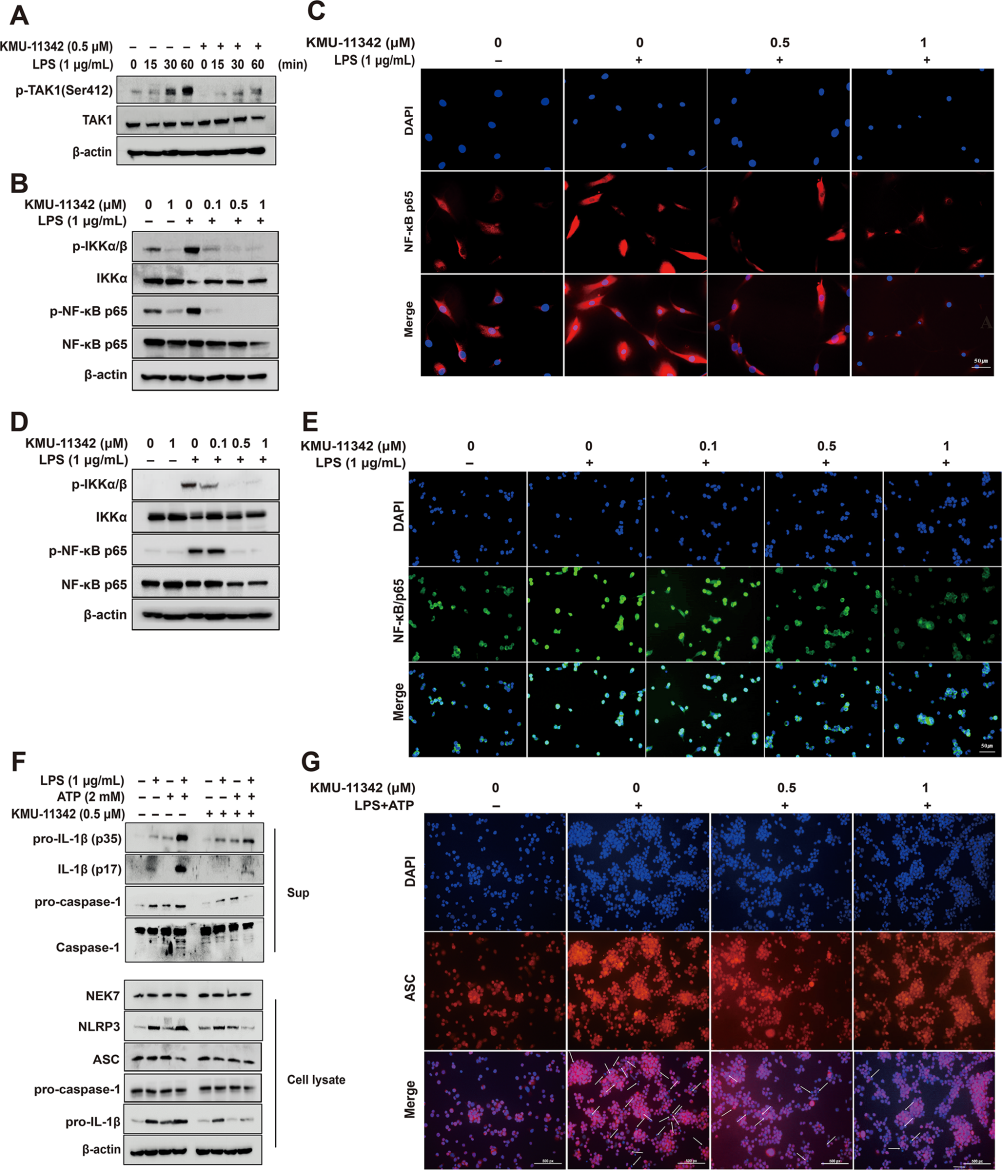
Inhibitory effect of KMU-11342 on activation of NF-κB signals and TAK1, MAPKs and NLRP3 inflammasome in human RA-FLS and THP-1 cells. RA-FLS and THP-1 cells were pre-treated with KMU-11342 for 1 h and stimulated with LPS (1 µg/mL) for 6 h. (**A**) THP-1 cells were stimulated with LPS (1 µg/mL) for the indicated time points. Whole cell lysates were isolated and used to measure the protein expression levels of p-TAK1 and TAK1 by Western blot analysis. (**B**) In human RA-FLS; whole cell lysates were isolated and used to measure the protein expression levels of p-IKKα/β, IKKα, p-NF-κB p65 and NF-κB p65 by Western blotting. (**C**) Human RA-FLS were stained with antibodies to NF-κB p65 (red) and DAPI (blue) and captured at ×200 using fluorescence microscope. (scale bar = 50 μm). (**D**) In THP-1 cells; whole cell lysates were isolated and used to measure the protein expression levels of p-IKKα/β, IKKα, p-NF-κB p65 and NF-κB p65 by Western blotting. (**E**) THP-1 cells were stained with antibodies to NF-κB p65 (green) and DAPI (blue) and captured at ×200 using fluorescence microscope. (scale bar = 50 μm). (**F**) THP-1 cells were stimulated with LPS (1 µg/mL) and ATP (2 mM) for additional 30 min. Cell lysate (Lysate) and media supernatant (Sup) were isolated and used to measure the protein expression levels of pro-IL-1β, IL-1β, pro-caspase-1, and caspase-1 for the Sup as well as NLRP3, ASC, pro-caspase-1, and pro-IL-1β for the Lysate by Western blot analysis. (**G**) The visualized ASC speck formation using immunofluorescence analysis with anti-ASC (red) antibody in THP-1 macrophages pretreated with KMU-11342 (0.5 and 1 µM) for 1 h followed by treatment with LPS (1 µg/ml) for 2 h and ATP (2mM) for 30 min. Arrows indicate ASC specks. DAPI (blue) was used as a nuclear marker. Scale bar: 50 μm

To explore the anti-inflammatory mechanisms of KMU-11342 in LPS-mediated inflammatory signaling pathways, we assessed the impact of KMU-11342 on the TAK1-NF-κB-NLRP3 inflammasome signaling cascade. KMU-11342 effectively inhibited the LPS-induced phosphorylation of TAK1 in THP-1 cells (Fig. 4A). Furthermore, pre-treatment with KMU-11342 in human RA-FLS and THP-1 cells curbed the LPS-induced phosphorylation of IKKα/β and NF-κB p65 (Fig. 4B, D). Additionally, immunofluorescence staining substantiated that KMU-11342 attenuated the LPS-mediated translocation of NF-κB p65 from the cytosol to the nucleus in both human RA-FLS and THP-1 cells (Fig. 4C, E). The activation of the NLRP3 inflammasome, a pivotal protein complex, plays significant role in the production, maturation, and secretion of the pro-inflammatory cytokine IL-1β. KMU-11342 demonstrated inhibitory effects on the up-regulation and maturation of IL-1β and the activation of caspase-1 induced by co-treatment with LPS and ATP, while protein expression levels of ASC and pro-caspase-1 remained unaltered (Fig. 4F). Additionally, KMU-11342 suppressed co-treatment with LPS and ATP-induced ASC oligomerization, forming large protein specks in the vicinity of the nucleus of THP-1 macrophages via immunofluorescence staining (Fig. 4G). These results suggest that KMU-11342 may inhibit the activation of NLRP3 inflammasome and the recruitment of ASCs, although it does not affect protein expression. Consequently, these findings illustrate that KMU-11342 impedes the LPS-mediated activation of the TAK1-NF-κB-NLRP3 inflammasome pathway.

### KMU-11342 attenuates receptor activator of nuclear factors κB ligand (RANKL)-induced osteoclast differentiation, up-regulation of osteoclastogenesis-associated factors, and activation of TAK1/MAPKs/NF-κB Signal pathways

**Fig. 5:**
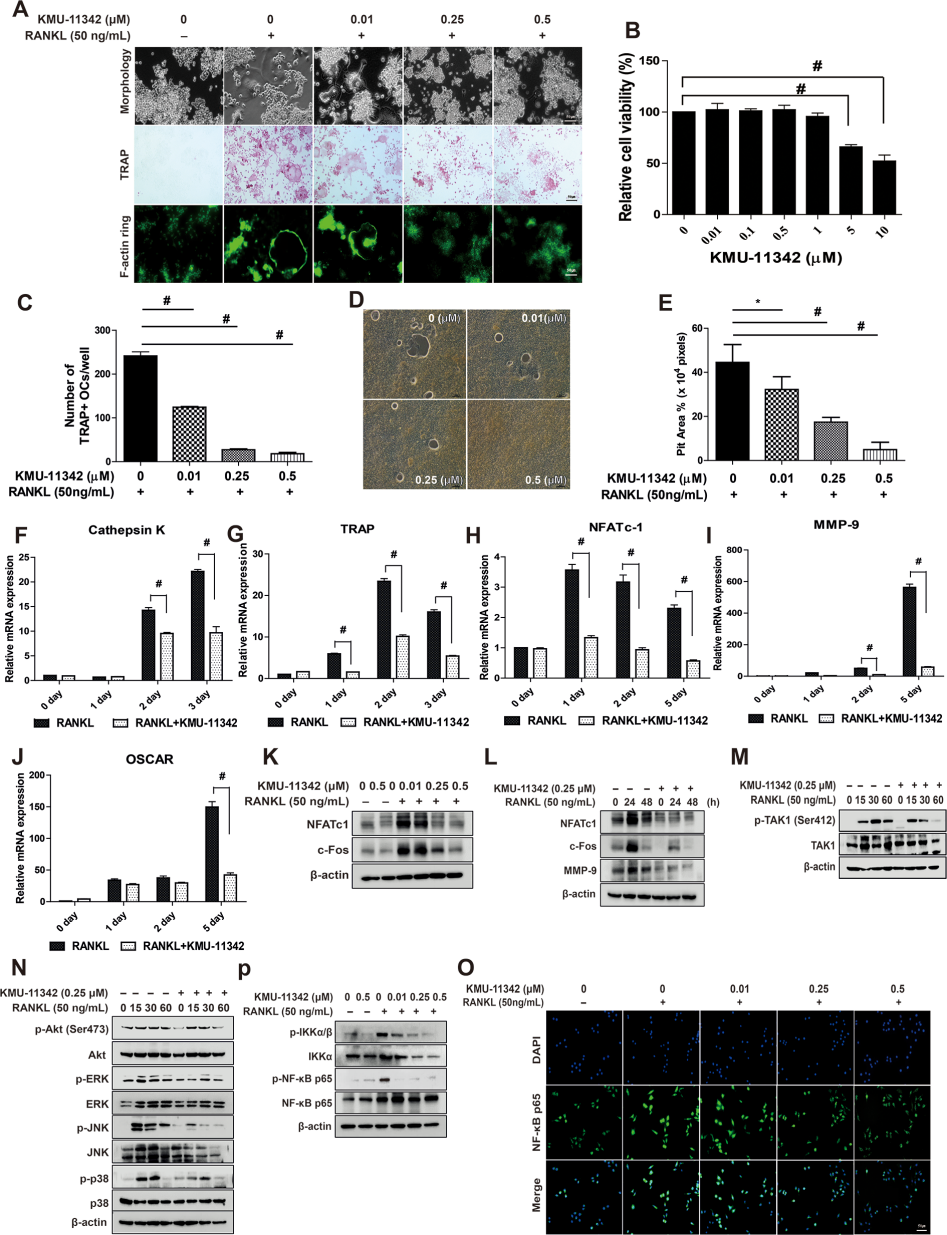
Inhibitory effect of KMU-11342 on RANKL-induced osteoclast differentiation in RAW264.7 cells. (**A**) Cells were pre-treatment with various concentrations of KMU-11342 (0.01, 0.25, and 0.5 µM) for 1 h, and made to undergo RANKL (50 ng/mL)-induced differentiation for 5 days. Each microscopic photograph depicts osteoclast differentiation cell morphology, stained with TRAP-stained osteclasts, and stained with rhodamine palloidin to show F-actin ring formation. (**B**) Cells were treated with different doses of KMU-11342 (0.01, 0.1, 0.5, 1, and 5 µM) for 24 h. Cytotoxic effect of KMU-11342 in RAW264.7 cells was performed by XTT assay. # *p* < 0.001 compared with control group. (**C**) The number of TRAP-positive multinuclear cells were counted. # *p* < 0.001 compared with RANKL only treated group. (**D**) Bone resorption assay. RAW264.7 cells were seeded into an osteo assay surface multiple well plate with 50 ng/mL RANKL plus various concentrations of KMU-11342 and were removed after 7 days. (**E**) The pit area (%) was determined Resorption planes from 5 random fields for each well using the imageJ program Values in the graph (**C, E**) indicate the mean ± SD of three independent experiments. * *p* < 0.05, # *p* < 0.001 compared with RANKL alone treated group. (**F–J**) RAW264.7 cells were treated KMU-11342 (0.25 µM) for 1 h and then stimulated with RANKL (50 ng/mL) for 1 to 5 days. Total RNA was extracted, and used to evaluate the mRNA expression levels of cathepsin K, TARP, NFATc-1, MMP-9, and OSCAR, respectively. # *p* < 0.001 compared with RANKL only treated group. (**K**) Cells were treated various concentration of KMU-11342 (0, 0.01, 0.25, and 0.5 µM) for 1 h and stimulated with RANKL (50 ng/mL) for 6 h. Whole cell lysates were isolated and used to measure the protein expression levels of NFAT-c1 and c-Fos by Western blot analysis. (**L**) Cells were treated various concentration of KMU-11342 (0.25 µM) for 1 h and stimulated with RANKL (50 ng/mL) for 24 to 48 h. Whole cell lysates were isolated and used to measure the protein expression levels of NFAT-c1, c-Fos, and MMP-9 by Western blot analysis. (**M–N**) Cells were stimulated with RANKL (50 ng/mL) for the indicated time points with or without 0.25 µM KMU-11342. (**M**) Whole cell lysates were isolated and used to measure the protein expression levels of p-TAK1 and TAK1 by Western blotting. (**N**) Whole cell lysates were isolated and used to measure the protein expression levels of p-Akt (Ser473), Akt, p-ERK, ERK, p-JNK, JNK, p-p38, and p38 by Western blot analysis. (**O**) Whole cell lysates were isolated and used to measure the protein expression levels of p-IKKα/β, IKKα, p-NF-κB p65 and NF-κB p65 by Western blotting. (**P**) Cells were stained with antibodies to the NF-κB p65 (green) and DAPI (blue) and captured at ×200 using fluorescence microscope. (scale bar = 50 μm)

The persistent progression of rheumatoid arthritis results in the inevitable destruction of bones and cartilage. So, we investigated whether the inhibitory effect of KMU-11342 on osteoclast differentiation, a process pivotal to bone resorption, and explore its influence on F-actin ring formation—an essential indicator of bone resorption. Osteoclast differentiation was induced in RAW264.7 cells using RANKL (50 ng/mL) over a period of 5 days. Immunofluorescence analysis demonstrated a dose-dependent suppression by KMU-11342 of the formation of TRAP-positive polynuclear osteoclasts and the extent of F-actin ring formation (Fig. 5A). Furthermore, KMU-11342 treatment significantly dose-dependently reduced the count of TRAP-positive osteoclasts (Fig. 5C) and exhibited no cytotoxicity up to a concentration of 0.5 µM in RAW264.7 cells (Fig. 5B). The bone resorption assays indicated that KMU-11342 decreased the formation of resorption pits in bone fragments (Fig. 5D), with a quantitative evaluation revealing a concentration-dependent reduction in the area of these pits (Fig. 5E). These findings suggest that KMU-11342 diminishes the bone resorption capability of osteoclasts. Subsequently, we assessed KMU-11342’s impact on osteoclast differentiation. KMU-11342 significantly attenuated RANKL-stimulated up-regulation of various osteoclastogenesis-associated factors, including cathepsin K, TRAP, osteogenic phosphatase (OSCAR), nuclear factor of activated T cells c1 (NFATc1), and MMP-9 (Fig. 5F–J). Furthermore, KMU-11342 inhibited the expression of c-Fos and NFATc1 protein levels—essential transcription factors in bone cell differentiation and production (Fig. 5K). MMP-9 protein expression was also suppressed by KMU-11342 treatment (Fig. 5L). To determine the mechanism underlying the inhibition of osteoclast differentiation by KMU-11342, we investigated the activation of TAK1, Akt, and MAPKs (ERK, JNK, and p38)—key components of RANKL-induced osteoclast differentiation. Remarkably, KMU-11342 inhibited RANKL-induced phosphorylation of TAK1, Akt, ERK, JNK, and p38 in RAW264.7 cells (Fig. 5M, N). Additionally, KMU-11342 strongly impeded RANKL-induced phosphorylation of IKKα/β and NF-κB (Fig. 5O). Immunofluorescence analysis further demonstrated that KMU-11342 suppressed the nuclear translocation of NF-κB p65 in RAW264.7 cells as observed under fluorescence microscopy (Fig. 5P). These results suggest that KMU-11342 inhibits osteoclast differentiation by targeting the Akt, ERK, JNK, p38, and the TAK1-NF-κB pathways.

### KMU-11342 upregulates the gene expression involved in mitochondria biogenesis pathway and increases mitochondrial DNA (mtDNA) copy number

**Fig. 6:**
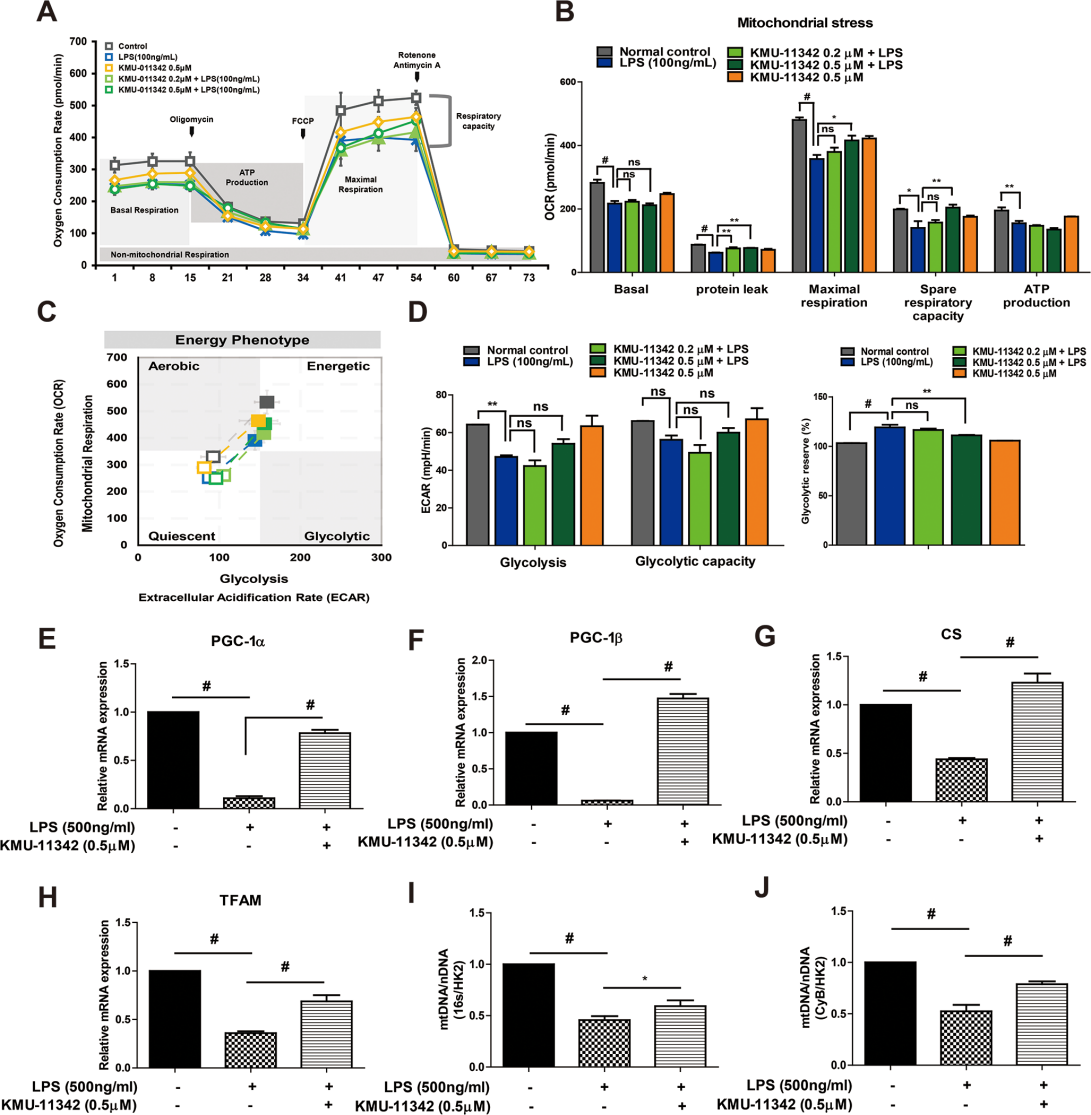
Effect of KMU-11342 in mitochondrial respiration and biogenesis. (**A–D**) RAW264.7 cells were pretreated with KMU-11342 (0.2, 0.5µM) for 1 h, and cells were stimulated with or without LPS (100ng/mL) for 2 h for Mito stress assay. (**A, B**) The respiratory profile of Oxygen consumption rate (OCR) and oxygen consumption rate for different aspects of mitochondrial respiration following exposure to KMU-11342 as determined using the Seahorse extracellular flux analyser (*n* = 3). (**C**) Energy map showing the evolution of mitochondrial respiration. Empty squares represent the energy phenotype under initial conditions, while filled squares represent energy demand conditions. The y-axis represents the OCR value of mitochondrial respiration, and the x-axis represents the ECAR value of the corresponding pathway. (**D**) Glycolysis in RAW264.7 cell determined from extracellular acidification rate (ECAR) by Seahorse assay, *n* = 3. (**E – J**) RAW264.7 cells pre-treatment with KMU-11342 for 1 h and stimulated with LPS (500ng/mL) for 6 h. (**E–H**) mRNA expression levels of PGC-1α, PGC-1β, CS, and TFAM were determined using real time-quantitative PCR. *P < 0.05, ** *P* < 0.01, # *p* < 0.001 (**I, J**) The relative mtDNA copy number estimated with qPCR measurement of 16 S, CyB, and HK2 level in LPS-treated RAW264.7 cell after 1 h of exposure to the 0.5 µM KMU-11342: 16 S/HK2 ratio and CyB/HK2 ratio

To determine the impact of KMU-11342 on energy metabolism during macrophage–mediated inflammatory responses, mitochondrial oxygen consumption rates were measured in macrophages using Seahorse extracellular analyzer. Inflammation induced by LPS compromised basal/maximal respiratory rates, protein leakage, spare respiration capacity, and mitochondrial ATP production (Fig. 6A). Conversely, KMU-11342 preserved mitochondrial function by promoting maximal cellular respiration (Fig. 6A, B). These results suggest that KMU-11342, known for its anti-inflammatory properties, also contributes to mitochondrial function preservation. The LPS-treated group displayed a metabolic shift from a quiescent state to aerobic pathway under stress conditions. However, the KMU-11342-treated group exhibited only a minor slight shift in energy metabolism from quiescent to an aerobic pathway (Fig. 6C). Moreover, the potential role of KMU-11342 in glycolysis regulation was explored by assessing it effect on glycolytic actibity in LPS-exposed macrophages. KMU-11342 was found to counteract the LPS-induced reduction in glycolysis and suppress glycolytic reserve in RAW264.7 macrophages (Fig. 6D). Subsequently, the influence of KMU-11342 mitochondrial biogenesis genes was investigated by examining genes related to this process, including Peroxisome proliferator-activated receptor gamma coactivator 1-alpha (PGC-1α), PGC-1β, citrate synthase (CS), and mitochondrial transcription factor A (TFAM). KMU-11342 markedly reversed the LPS triggered down-regulations of genes associated with mitochondrial biogenesis (Fig. 6E–H). Furthermore, the study evaluated the association between KMU-11342 treatment and changes in total mitochondrial mass, revealing that KMU-11342 alleviated the LPS-induced decrease in the mtDNA copy numbers ratio (Fig. 6I, J).

## Discussion

Rheumatoid arthritis (RA) stands as a prevalent, persistent autoimmune disorder marked by chronic inflammation of the joints, frequently leading to gradual joint deterioration and functional impairment [1]. Although various drug have been developed to counteract RA [8], the quest for innovative medications continues due to the limited efficacy of adverse effects of existing treatments [9]. A deeper understanding of RA pathogenesis is crucial for enhancing treatment effectiveness, particularly in managing pathological aspects such as bone erosion and regulating the inflammatory signaling pathways of implicated immune cells [9]. In this study, we introduced a novel multi-protein kinase inhibitor, KMU-11342, showcasing its potential in mitigating inflammation in various immune cells implicated in RA pathogenesis and in inhibiting osteoclastogenesis. Additionalyy, KMU-11342’s drug development prospects are further supported by its demonstrated low cytotoxicity in vivo studies using zebrafish and its electrophysiological safety confirmed through ex vivo studies on rat hearts. KMU-11342, a 3,5-disbustituted indolin-2-one derivative of 3-((1*H*-imidazol-5-yl)methylene)-5-(6-(cyclopentylamino)pyrazin-2-yl)indolin-2-one, emerged from screening our proprietary kinase inhibitor library for anti-inflammatory. The indolin-2-one scaffold is pivotal in kinase. Inhibitor discovery, as evidenced by its role in the development of analog such as sunitinib and nintedanib, used in cancer and idiopathic pulmonary fibrosis treatments, respectively [10]. Morever, indolin-2-one-based anti-inflammatory inhibitors have been reported in recent literature [11–14]. Significantly, KMU-11342 exhibited potent inhibition of protein kinases associated with inflammation (Table 2). Cardiovascular toxicity is remains pivotal challenge in the development and clinical application of drugs, including kinase inhibitors [15]. Consequently, a thorough assessment of potential torsade de Points (TdP), a severe form of ventricular arrhythmia associated with delayed ventricular repolarization is crucial for ensuring the safety of new therapeutics [16, 17]. The international conference on Harmonisation (ICH) has released S7B and E14 guidelines for comprehensive cardiotoxicity evalutions, such as LVP and ECG to assess the risk of arrhythmia induction [17, 18]. In this study, the electrophysiological safety of KMU-11342 was validated through ECG, and LVP analyses (Fig. 1A, B). While traditional models for drug toxicity evaluation have predominantly used higher animals zebrafish have emerged as a viable vertebrate model for initial drug screening, assessing developmental and general toxicity [19]. Consequently, zebrafish were employed in this study to ascertain the developmental toxicity of KMU-11342. The results indicated minimal cardiotoxicity and non-lethal effects on zebrafish embryos, including the absence of pathological changes in major organs during development. Recent evidence spanning over the past decade emphasizes the critical role of Toll-like receptors (TLRs) in the onset of various rheumatic diseases [20]. Reports have particularly highlighted the involvement of TLRs in RA pathogenesis [21] with TLR4 being notably overexpressed in the inflamed synovium of RA patients [22]. TLR4 signaling is mediated through the recruitment of specific adaptor molecules and occurs via pathways including mitogen-activated protein kinases (MAPKs), nuclear factor kappa B (NF-κB), and janus family tyrosine kinase (JAK)-STAT [23–25]. Understanding and modulating these pathways thereby controlling their overactivation can significantly contreibute to the treatment of inflammatory diseases. Accordingly, we investigated the anti-inflammatory mechanisms of KMU-11342 within an LPS-induced TLR4-mediated inflammatory activation. Prostaglandins (PGs), derived from arachidonic acid, are lipid mediators of inflammation that play significant role in the pathogenesis of RA and are considered potential therapeutic targets [26]. COXs are crucial enzymes in synthesizing PGH_2_, the precursor of various PGs. There are two primary isoforms in mammals, COX-1 and COX-2 [27]. Significantly, numerous studies have shown elevated expression of COX-2 in the synovial tissues of RA patients [28–30]. Furthermore, COX-2 selective inhibitors represent a novel therapeutic approach, offering effectively control of inflammation and pain with fever side effects, such as gastrointestinal irritation [31]. Additionally, the NOS/nitric oxide signaling pathway has been increasingly implicated in RA pathogenesis [32]. KMU-11342 demonstrated inhibition of LPS-induced up-regulation of COX-2 and iNOS in both human RA-FLS and THP-1 cells (Fig. 2A-F). MAPKs are pivotal regulators of pro-inflammatory cytokine synthesis and are present in the synovial tissues of RA patients [33]. TAK1, and MAP3K7, key mediators in innate and adaptive immune responses, integrate inflammatory signals mediated by cytokines, TLR, and T and B cell receptors [34, 35]. In this investigation, KMU-11342 was observed to inhibit the LPS-induced phosphorylation of JNK and TAK1 in Figs. 2G and 4A, respectively. These findings suggesting its potential as an anti-inflammatory agent targeting key inflammation regulators.

Recent developments in anti-inflammatory pharmacotherapy have introduced drugs targeting diverse inflammatory signaling pathways [36]. Methotrexate, for instance, has been a cornerstone in RA treatment since the 1980s. Agents blocking pro-inflammatory cytokines, such as TNF-α inhibitors (adalimumab, etanercept, certolizumab, infliximab, and golimumab), interleukin-1 (IL-1) antagonists (anakinra), and IL-6 inhibitors (toxylizumab), has become a standard practice [37]. Moreover, tofacitinib, a small molecule inhibitor targeting JAK1, JAK3, and JAK2, marked a significant milestone as the kinase inhibitor approved in the United States [38]. However, the long-term administration of biological agents for RA poses increased risks of cancer and infections, along with drug reactions and various other issues associated with drug resistance and chronic use. These challenges underscore the necessity for further drug development in RA treatment. KMU-11342 exhibited superior anti-inflammatory efficacy compared to celecoxib, ibrutinib, methotrexate, and tofacitinib (Fig. 3B). While additional research is essential, these findings position KMU-11342 as a promising drug candidate for RA treatment. RA manifests as a systemic inflammatory joint disorder, characterized by the intricate interplay of pathophysiological processes encompassing the activation of RA-FLS, osteoclastogenesis, and synovial membrane inflammation [39]. Notably, FLS contributes to many pathological aspects of RA, such as synovitis, pannus growth, and ultimately cartilage/bone destruction, by promoting them [40]. Pro-inflammatory cytokines and chemokines, integral to TLRs signaling, intricately contribute to the pathogenesis of RA, serving as pivotal mediators released by RA-FLS in response to cytokines and TLR ligands [21, 40]. Therefore, controlling FLS could be a promising target for treating RA, as it may lead to an appropriate therapeutic effect for the disease. As shown in Fig. 3C – F, in human RA-FLS, KMU-11342 inhibited the LPS-induced production of various chemokines, such as CXCL10, CCL2, CCL3, and CCL4, which are highly expressed in RA [41]. Moreover, macrophages central to RA pathophysiology, produce pro-inflammatory cytokines that contribute to chronic inflammation, tissue damage, and pain in affected joints [42]. KMU-11342 effectively suppressed the LPS-induced upregulation of IL-1β, IL-6, and TNF-α in both RA-FLS and THP-1 cells. These findings strongly indicate the anti-inflammatory properties of KMU-11342. NF-κB, a nuclear transcription factor, orchestrates the expression of diverse inflammatory mediators, encompassing inducible enzymes like COX-2 and iNOS, as well as TNF-α, IL-1β, IL-6, and various cell-adhesion molecules [43]. As shown in Fig. 4B – E, KMU-11342 effectively curtailed LPS-induced phosphorylation of IKKα/β and impeded the phosphorylation and subsequent nuclear translocation of NF-κB p65 in both RA-FLS and THP-1 cells. NLRP3 expression, regulated by NF-κB binding to the NLRP3 promoter [44], and the NLRP3 inflammasome, implicated in RA development, play a pivotal role in caspase-1 activation, which mediates the maturation and secretion of IL-1, a pro-inflammatory cytokine [45, 46]. KMU-11342 attenuated the synergistic effect of LPS and ATP on caspase-1 activation and NLRP3 upregulation in THP-1 cells (Fig. 4F). When NLRP3 inflammasome is activated, the adapter protein ASC assembles into large protein complexes known as ASC specks or pyroptosomes [47], which is considered a typical hallmark of inflammasome activation. In other words, the activation of NLRP3 inflammasome is essential for the oligomerization of ASC, which generates speck formation [48]. As shown in Fig. 4G, KMU-11342 inhibited inflammasome activation as represented by ASC speck formation. These findings suggest that KMU-11342, inhibiting NF-κB activity, holds potential as a therapeutic agent for the RA treatment.

RA is marked by the deterioration of joint cartilage, bone, and persistent inflammatory synovitis [49]. RA synovial tissue secretes RANKL, a pivotal cytokine for osteoclast differentiation. Its formation and activation are mediated through the RANKL/RANK pathway [50, 51]. Osteoclast differentiation via this pathway is controlled by transcription factors such as c-fos, NF-κB, and NFATc1 [52]. Specifically, NFATc1 governs osteoclast-specific genes like MMP-9 [53] and TRAP [52], and it cooperatively regulates Cathepsin K alongside p38 [54].

Additionally, the osteoclast-associated receptor OSCAR plays a crucial role in RANKL-induced osteoclast differentiation [55]. In this study, KMU-11342 inhibited RANKL-induced multinucleated cell differentiation, indicative of osteoclast differentiation (Fig. 5C), and the formation of F-actin rings, critical for osteoclast-mediated bone resorption [56] (Fig. 5A).

It also suppressed the RANKL-induced enhancement of NFATc-1 and MMP-9 mRNA and protein levels (Fig. 5H, I, K, L) and decreased Cathepsin K, TRAP, and OSCAR mRNA levels (Fig. 5G, J), which are specific markers of osteoclast differentiation. Furthermore, KMU-11342 impeded the phosphorylation of TAK1, a precursor of NF-κB, vital for RANKL-induced osteoclastogenesis (Fig. 5M). Notably, the stimulation of TLR and pro-inflammatory cytokines within the MAPK pathway influences bone cell adhesion, migration, fusion, survival, and bone resorption [57]. As depicted in Fig. 5N, KMU-11342 mitigated the RANKL-induced phosphorylation of ERK, JNK, and p38. Mitochondria serve as an important site of intracellular energy production, and the effects of drugs on mitochondrial function are relevant in determining drug efficacy and toxicity [58]. Moreover, mitochondrial biogenesis is orchestrated by master regulators encoded genes of the nucleus and mitochondria, such as PGC-1α, PGC-1β, and TFAM, which mediate mtDNA replication [59]. In particular, PGC-1α is a major regulator of mitochondrial biogenesis, and While TFAM is primarily expressed within the nucleus, it exerts its regulatory actions within the mitochondrial compartment [60]. And the mtDNA copy number frequently serves as a reliable marker for assessing mitochondrial content [61]. Under the conditions of LPS-induced inflammatory response, the administration of KMU-11342 resulted in both a decrease and an increase in the levels of GC-1, CS, and TFAM. Furthermore, KMU-11342 demonstrated the ability to elevate DNA copy number. These findings strongly imply that KMU-11342 exerts a stimulating effect on mitochondrial biogenesis. In extensive investigations to date, the identification of pivotal therapeutic targets crucial for the clinical efficacy of targeted drugs in rheumatoid arthritis (RA), including cytokines with diverse and multifaceted expression activities and kinases involved in upstream immune synapses or signaling pathways, holds significance [62]. Our study strongly asserts that KMU-11342, despite being a multifunctional kinase, exhibits substantial potential for the treatment of RA.

## Conclusion

In summary, this study explored the anti-rheumatic properties of KMU-11342 by inhibiting the TLR4/NF-κB/NLRP3 inflammasome signaling in LPS-stimulated RA-FLS and THP-1 cells, and by attenuating osteoclast differentiation. Importantly, KMU-11342 exhibited minimal in vivo cytotoxicity and ex vivo electrophysiologic cardiotoxicity. Hence, the findings suggest that KMU-11342 holds promise as a prospective therapeutic drug for rheumatoid arthritis.

## Data Availability

No datasets were generated or analysed during the current study.
